# Deriving percentage study weights in multi-parameter meta-analysis models: with application to meta-regression, network meta-analysis and one-stage individual participant data models

**DOI:** 10.1177/0962280216688033

**Published:** 2017-02-06

**Authors:** Richard D Riley, Joie Ensor, Dan Jackson, Danielle L Burke

**Affiliations:** 1 Research Institute for Primary Care and Health Sciences, Keele University, UK; 2 MRC Biostatistics Unit, Cambridge Institute of Public Health, Forvie Site, Robinson Way, Cambridge Biomedical Campus, Cambridge, UK

**Keywords:** Percentage study weights, Fisher’s information, individual patient data meta-analysis, meta-regression, network meta-analysis

## Abstract

Many meta-analysis models contain multiple parameters, for example due to multiple outcomes, multiple treatments or multiple regression coefficients. In particular, meta-regression models may contain multiple study-level covariates, and one-stage individual participant data meta-analysis models may contain multiple patient-level covariates and interactions. Here, we propose how to derive percentage study weights for such situations, in order to reveal the (otherwise hidden) contribution of each study toward the parameter estimates of interest. We assume that studies are independent, and utilise a decomposition of Fisher’s information matrix to decompose the total variance matrix of parameter estimates into study-specific contributions, from which percentage weights are derived. This approach generalises how percentage weights are calculated in a traditional, single parameter meta-analysis model. Application is made to one- and two-stage individual participant data meta-analyses, meta-regression and network (multivariate) meta-analysis of multiple treatments. These reveal percentage study weights toward clinically important estimates, such as summary treatment effects and treatment-covariate interactions, and are especially useful when some studies are potential outliers or at high risk of bias. We also derive percentage study weights toward methodologically interesting measures, such as the magnitude of ecological bias (difference between within-study and across-study associations) and the amount of inconsistency (difference between direct and indirect evidence in a network meta-analysis).

## 1 Introduction

Meta-analysis is the synthesis of quantitative information from related studies to produce summary (pooled) results to help answer clinically relevant questions, such as whether a treatment is effective. Statistical models for meta-analysis often use aggregate data (such as a treatment effect estimate and its variance) from each study, but increasingly they utilise individual participant data (IPD).^1,2^ Regardless of the approach taken, forest plots are an important way to disseminate results to a clinical audience as they quickly summarise the size and spread of individual study results alongside the summary meta-analysis result. In relation to forest plots, the PRISMA Statement recommends that ‘… it is preferable also to include, for each study, the numerical group-specific summary data, the effect size and confidence interval, and *the percentage weight*’.^3^ Percentage study weights aim to break down the summary meta-analysis result into the relative contribution of each individual study, and are easily interpretable by non-statisticians.

In this article, we propose a general approach to deriving percentage study weights in meta-analysis models with multiple parameters, including meta-regression, network (multivariate) meta-analysis and IPD models with interactions. Our approach includes, as a special case, the simpler, traditional situation where just a single parameter (such as a treatment effect) is to be synthesised. However, most meta-analysis models contain multiple parameters, for example due to multiple outcomes, multiple treatments or multiple regression coefficients. In particular, meta-regression models may contain multiple study-level covariates,^4^ and one-stage IPD meta-analysis models may contain multiple patient-level covariates and interactions.^5,6^

Our aim is to allow researchers to produce percentage study weights for each parameter of interest within a multi-parameter meta-analysis model. Our motivation is to increase transparency of each study’s contribution, which is especially important when some studies are potential outliers or at high risk of bias, to reveal how much their data contributed to the overall meta-analysis result. Similarly, if clinical decision makers are concerned that a meta-analysis result may be unreliable for translation to practice due to the inclusion of a study from a different clinical population or setting, then knowing the study’s contribution is important. Percentage study weights are also helpful for methodological purposes, for example to: (a) help understand and explain differences between one- and two-stage IPD meta-analysis results, for which there is much interest;^7–11^ (b) help understand differences between results from IPD meta-analyses and those based on aggregate data obtained from published studies, which may include different studies and/or patients;^12–14^ (c) to ascertain the contribution of IPD studies in meta-analyses that combine IPD and non-IPD (aggregate data) studies;^15,16^ (d) understand the contribution of small studies in meta-analyses with funnel plot asymmetry (i.e. with potential publication or availability bias)^17^ or (e) to understand which studies are contributing most toward potential bias or inconsistency terms, for example within a network meta-analysis.^18^

The article is structured as follows. Section 2 recaps percentage study weights in a traditional meta-analysis of a single parameter, and reveals how they can be expressed on the variance scale using a decomposition of Fisher’s information. In Section 3, we extend this concept to multiple parameter models expressed within a general or generalised linear mixed model framework. These include one-stage IPD models, meta-regression and multivariate (network) meta-analysis. Section 4 provides three applied examples, covering IPD meta-analysis, meta-regression and network meta-analysis, for the estimation of treatment effects and treatment-covariate interactions. Special consideration of weights toward bias and inconsistency terms is also given. Section 5 concludes with discussion.

## 2 Derivation of percentage study weights in a traditional, single-parameter meta-analysis

We begin by outlining percentage study weights within a traditional meta-analysis that uses a two-stage approach to summarise a single parameter. We focus on summarising a treatment effect from multiple randomised controlled trials, but the concepts are immediately applicable to a different parameter of interest, such as a treatment-covariate interaction.

### 2.1 First stage

Let us assume that there are *i* = 1 to *K* randomised trials for meta-analysis, each comparing a particular treatment to control, and that the treatment effect is of interest. In the two-stage approach, the first stage involves obtaining (e.g. from study publications or by using IPD) the *K* treatment effect estimates (θ^i) and their within-study variances (var(θ^i)). Let us assume IPD are available for all trials, so that we can analyse the raw data for each trial separately. The choice of analysis model depends on the outcome data type.^5^ For example, for continuous outcome responses, such as blood pressure, a linear regression model might be fitted,^19^ potentially adjusting for baseline values (analysis of covariance, ANCOVA),^20^

(1) $$\begin{array}{c} y_{\text{F}\mathrm{ij}}=\varphi_{i}+\lambda_{i}y_{\text{B}\mathrm{ij}}+\theta_{i}x_{\mathrm{ij}}+e_{\mathrm{ij}}\\ e_{\mathrm{ij}}∼N(0,\sigma_{i}^{2}) \end{array}$$

where $y_{\text{F}\mathrm{ij}}$ and $y_{\text{B}\mathrm{ij}}$ denote the final (F) and baseline (B) value, respectively, for patient *j* in trial *i*, and $x_{\mathrm{ij}}$ is 0/1 for participants in the control/treatment group, and $e_{\mathrm{ij}}$ is the residual variance, assumed normally distributed with mean zero and variance $\sigma_{i}^{2}$. Thus, $\varphi_{i}$ is the intercept, $\lambda_{i}$ is the effect of a one-unit increase in baseline blood pressure and $\theta_{i}$ is the treatment effect. Estimation of model (1) gives β^i = (φ^i, λ^i θ^i) and also σ^i2. Taking the latter as known, we can invert Fisher’s observed information matrix for β^i (i.e. invert the variance of the score for β^i) to estimate the variance matrix for β^i:

var(β^i)=(var(φ^i)cov(φ^i,λ^i)cov(φ^i,θ^i)cov(φ^i,λ^i)var(λ^i)cov(λ^i,θ^i)cov(φ^i,θ^i)cov(λ^i,θ^i)var(θ^i))

### 2.2 Second stage

In the second stage, traditionally only a single parameter estimate from each study is of interest, such that a univariate meta-analysis is needed. In the above example, the treatment effect estimates (θ^i) are the focus, and so a meta-analysis model is needed to combine these across trials. A fixed effect model assumes that the underlying treatment effect is the same in all studies, and thus the θ^i values are all estimates of a common treatment effect, θ. The model can be written as:

(2) θ^i=θ+ɛiɛi∼N(0,var(θ^i))

In model (2), the var(θ^i) estimates are usually assumed known,^21^ and the model can be fitted using maximum likelihood to give the summary estimate, θ^, of the *common* treatment effect. A random effects meta-analysis model allows for between-study heterogeneity in the true treatment effect, and can be written as,

(3) θ^i=θi+ɛiθi=θ+uiui∼N(0,τ2)ɛi∼N(0,var(θ^i))

where the var(θ^i) estimates are again typically assumed known, and *u_i_* denotes a random effect that indicates the treatment effect in the *i*th trial, $\theta_{i}$, is assumed normally distributed about an average treatment effect, θ, with between-study variance, $\tau^{2}$. Model (3) can be estimated using, for example, method of moments^22^ or restricted maximum likelihood, to give the summary estimate, θ^, of the *average* treatment effect across trials. Extension of model (3) to a meta-regression within study-level covariates will be considered in Section 3.

### 2.3 Decomposing Fisher’s information for θ^

The maximum likelihood solution for the summary meta-analysis estimate (θ^) is

(4) θ^=∑i=1Kwiθ^i∑i=1Kwi

where wi=(var(θ^i))-1 in the fixed effect model, and wi=(var(θ^i)+τ^2)-1 in the random effects model. The summary estimate, θ^, is thus a weighted average of the θ^i, with the study weights (*w_i_*) depending on the ‘known’ sampling error (var(θ^i)) and, in the random effects setting, also the estimated between-study variance (τ^2).

The variance of θ^ is var(θ^) = 1/$\sum_{i=1}^{K}w_{i}$. This is obtained by the inverse of Fisher’s observed information matrix which, as models (2) and (3) contain only one main parameter (θ), is just a scalar (i.e. a 1 by 1 matrix) equal to $\sum_{i=1}^{K}w_{i}$. The total observed information (Itotal(θ^), say) toward θ^ can be written as,

(5) Itotal(θ^)=(var(θ^))-1=∑i=1Kwi=∑i=1KIi(θ^)

where Ii(θ^) is used to denote the information attributed to study *i*. As studies are independent, the total information is simply the sum of the Ii(θ^). This decomposition will be utilised again in Section 3, as it generalises to more complex meta-analysis models. The Ii(θ^) can be computed by re-fitting the meta-analysis model but with just study *i* included, whilst holding remaining variance estimates at their values from the full analysis (i.e. hold var(θ^i) and, in the random effects model, also τ^).

Interestingly, we can now re-express the variance of θ^ into a linear sum of independent study terms by utilising the decomposition of Fisher’s information matrix, as follows:

(6) var(θ^)=var(θ^)×Itotal(θ^)×var(θ^)=var(θ^)×∑i=1KIi(θ^)×var(θ^)=var(θ^)×∑i=1Kwi×var(θ^)=∑i=1Kvar(θ^)×wi×var(θ^)=∑i=1KWi

Thus, we have Wi=var(θ^)×wi×var(θ^)=var(θ^)×Ii(θ^)×var(θ^), and these *W_i_* allow us to derive study weights on the variance scale, as now described.

### 2.4 Derivation of percentage study weights

Given the meta-analysis solution of (4), it is well-known that the corresponding percentage study weights are obtained by:

$$\%\mathrm{weight}\text{ of study}i=100\%\times \frac{w_{i}}{\sum_{i=1}^{K}w_{i}}$$

Thus, those studies with the largest *w_i_* values will have the largest weight. Following equation (6), we can equivalently consider percentage weights in terms of *W_i_* by:

(7) %weight of studyi=100%×Wi∑i=1KWi=100%×Wivar(θ^)

Therefore, the well-known percentage weights for a single parameter meta-analysis are equivalent to a decomposition of the variance, var(θ^), into the sum of independent *W_i_* terms. This concept is utilised in Section 3 to extend percentage study weights to more complex, multi-parameter meta-analysis models. Indeed, the traditional use of $100\%\times w_{i}/\sum_{i=1}^{K}w_{i}$ to define percentage weights actually works on the precision scale, which does not generalise to a multi-parameter meta-analysis model. However, the use of the variance scale does generalise, and so use of *W_i_* will become increasingly important.

## 3 Derivation of percentage study weights in multi-parameter meta-analysis and meta-regression models

We now use the concept of decomposing the variance of parameter estimates to derive percentage study weights in more complex, multi-parameter models. We begin with one-stage IPD models with continuous outcomes via linear mixed models, before then extending to meta-regression, multivariate meta-analysis and generalised linear mixed models. We focus on random effects models, as fixed effect models are merely a simplified case.

### 3.1 One-stage meta-analysis using linear mixed models

#### 3.1.1 Model specification and estimation

A one-stage IPD meta-analysis model for continuous outcomes can be expressed within a linear mixed model in matrix form, using a design matrix, **X**, for the fixed effects, β, and a design matrix, **Z**, for the random effects, **u**:^23,24^

(8) Y=Xβ+Zu+e

Here **Y** is a column vector containing all the continuous response values from all the participants from all studies, with length therefore equal to the total number of participants across studies. **β** is a column vector containing all the unknown fixed (mean) effects in the model, **u** contains the study-level random effects (typically assumed to follow a multivariate normal distribution with mean vector **0** and variance matrix **G**), and **e** contains the participant-level residuals (typically assumed to follow a multivariate normal distribution with mean vector **0** and variance matrix **R**). A detailed example is given in the supplementary material 1(a), for a one-stage ANCOVA meta-analysis model with a random treatment effect.^20^

Let **V** be the variance of **Y** conditional on **X**, which can be expressed as:^23^

(9) $$V=\text{var}(Y)=\text{var}(X\beta +\mathrm{Zu}+e)={\mathrm{ZGZ}}^{T}+R$$

The supplementary material 1(a) gives the specification of **V** for a one**-**stage ANCOVA model (also see the example in Section 4.1). The generalised least squares procedure minimises $(Y-X\beta {)}^{T}V^{-1}(Y-X\beta )$ with respect to β by differentiating with respect to β and setting the first derivative to zero; this gives the well-known solution of β^=(XTV-1X)-1XTV-1Y.^23,24^ Note that **V** must also be estimated simultaneously alongside β, through an iterative procedure until convergence is achieved. However, here we assume **V** is considered ‘known’ when used within the solution for β^, and thus **V** is usually replaced with its estimate.

The estimated variance matrix of β^ can be obtained as the inverse of Fisher's observed information matrix by var(β^)=(XTV-1X)-1, where again **V** is usually replaced with its estimate and assumed known. Each diagonal element of $(X^{T}V^{-1}X{)}^{-1}$ gives the estimated variance of one of the parameter estimates in the model. For instance, for the ANCOVA example in the supplementary material 1(a), $(X^{T}V^{-1}X{)}^{-1}$ is a 7 by 7 matrix, and the element (7,7) of the matrix gives var(θ^), the variance of the treatment effect, θ^.

#### 3.1.2 Decomposing Fisher’s information matrix

Let var(β^)=(XTV-1X)-1 be the estimated variance matrix of β^ after a one-stage IPD meta-analysis including all participants from all trials, with the diagonal elements for this matrix giving the corresponding variance of each parameter estimate (e.g. var(θ^)). Fisher’s observed information matrix for β^ is Itotal(β^)=(var(β^))-1=(XTV-1X).

We now generalise the decomposition outlined in Section 2.3. That is, we decompose Fisher’s information matrix for β^ into the sum of study-specific information matrices, using Itotal(β^)=∑i=1KIi(β^). This assumes the meta-analysis contains *independent studies*, and thus, although we give the general form of model (8) above, we are only focusing on meta-analysis with independent studies.

To obtain Ii(β^), we suggest re-computing Fisher’s information for β^ after removing the participants in all but the *i*th study, whilst keeping the remaining elements of **X** and **V** exactly as specified/estimated in the full meta-analysis of all studies. This extends the observation noted under equation (5) that Ii(θ^) can be computed by re-fitting the meta-analysis model but with just study *i* included, whilst holding remaining variance estimates at their values from the full analysis. Therefore, the user must specify and derive,

(10a) Ii(β^)=(XiTVi-1Xi)

where $X_{i}^{T}$ is the reduced design matrix containing rows just for participants in study *i*, and $V_{i}$ is the corresponding reduced variance matrix with entries as estimated in the full analysis.

An alternative approach is to derive Ii(β^) using,

(10b) Ii(β^)=(XTV∇i-1X)

where **X** is as specified in the full analysis and $V_{\nabla (\text{i})}$ is held to be the same as **V** in the full analysis, but for those patients not in study *i* the associated diagonal elements of **V** are replaced with a very large number (e.g. 1000000000) and all associated covariance terms set to zero (thus, the ‘∇i’ notation denotes the elements of **V** have changed for all participants except those in study *i*). This data augmentation approach ensures that participants external to study *i* have negligible contribution toward Ii(β^). Appendix 1 shows how SAS Proc Mixed can be used for this purpose, capitalising on the ‘parms’ statement;^24^ it also conveniently invokes the fast SAS estimation procedure to derive Ii(β^), and thus the user avoids needing to specify design and variance matrices themselves.

#### 3.1.3 Derivation of percentage study weights

We now propose how to derive percentage study weights in one-stage, multi-parameter IPD models by generalising the approach outlined in equations (6) and (7) for single parameter meta-analysis models. Recall that in equations (6) and (7), we decomposed the total variance of a parameter estimate into the sum of independent *W_i_* terms (Wi=var(θ^)×Ii(θ^)×var(θ^)), and used this to derive percentage weights by comparing each *W_i_* with the total variance. For one-stage models, we now have a multi-parameter situation, and so aim to decompose the variance matrix (var(β^)) into the sum of independent weight matrices, Wi(β^) say. This can be achieved by generalising equation (6) by utilising the decomposition of Fisher’s information matrix, as follows:

(11) var(β^)=var(β^)×Itotal(β^)×var(β^)=var(β^)×∑i=1KIi(β^)×var(β^)=∑i=1KWi(β^)

Equation (11) uses the variance scale (left-hand side), whilst utilising (within the right-hand side) the property that the total information matrix can be decomposed into the sum of the Ii(β^). This is consistent with the framework for weights in the simpler, single-parameter meta-analysis model (as derived in equation (6)), and follows the same decomposition of the variance as shown for multivariate meta-analysis by Jackson et al.^25^ It therefore forms the basis of our proposal for deriving percentage study weights in all meta-analysis models containing independent studies. It provides study-specific weight matrices (Wi(β^)=var(β^)×Ii(β^)×var(β^)), and these sum to give the total variance matrix for β^.

For each parameter estimate within β^, percentage study weights can now be derived by comparing the corresponding diagonal entries of Wi(β^) and var(β^). So, if the parameter corresponding to row *r* of β^ is of interest, we can derive

(12) %weight of studyi=100%×Wi(β^)r,r∑i=1KWi(β^)r,r=100%×Wi(β^)r,rvar(β^)r,r

where the ‘r,r’ notation refers to the element (r,r) of the corresponding matrix. For the ANCOVA example in the supplementary material 1(a), there are seven parameter estimates and so each Wi(β^) is a 7 by 7 matrix; the treatment effect parameter (θ) is the seventh, and so the values of Wi(β^)7,7 and var(β^)7,7 are needed to derive the percentage study weight toward the treatment effect estimate, θ^.

It is important to note that the diagonal elements of each Wi(β^) can be different. Therefore, the percentage weight of study *i* may differ for each parameter in the model, and so should be reported separately.

### 3.2 Meta-regression models

Meta-regression models extend model (3) by including study-level covariates, and could be applied in the second stage of a two-stage approach. Such models are themselves general linear models containing multiple parameters, and so percentage study weights toward a parameter can be derived akin to the approach described above. That is, one needs to:

- fit the meta-regression and obtain var(β^),
- derive Ii(β^) using either equation (10a) or (10b), which corresponds to Fisher’s information matrix from a ‘meta-regression’ of just study *i*, with within and between-study variances held fixed at their values from the full analysis,
- derive Wi(β^)=var(β^)×Ii(β^)×var(β^),
- calculate percentage study weights for each parameter using equation (12).

An example is given in Section 4.2.

### 3.3 Multivariate meta-analysis and network meta-analysis models

A multivariate meta-analysis or multivariate meta-regression model jointly synthesises multiple correlated effects simultaneously (e.g. for multiple outcomes), whilst accounting for their correlation.^26^ Typically, they assume a multivariate normal distribution within and between studies, and thus are a multiple outcome extension of univariate models (2) and (3). As such, they also fall within the linear mixed model framework of equation (8),^27^ and so percentage study weights in multivariate models can also be derived using our proposal based on equations (11) and (12). Indeed, these will produce percentage weights that correspond exactly to those proposed by Jackson et al. for multivariate meta-analysis and implemented within the ‘mvmeta’ module of Stata.^28^ Jackson et al. derive their percentage weights by utilising a decomposition of the score statistic, but this reduces to the same proposal as in equation (12).

Multivariate meta-analysis is illustrated further in Section 4.3, in the context of a network meta-analysis of multiple treatment comparisons. Like an IPD meta-analysis, a network meta-analysis can be conducted as either a two- or a one-stage approach. The two-stage approach first derives treatment effect estimates (contrasts) between pairs of treatments in each study (together with their variances and correlations), and then synthesises them in a multivariate meta-analysis model.^18^ This can be implemented in, for example, the Stata module ‘network’,^29^ and provides percentage weights according to the approach of Jackson et al.^25^ An alternative one-stage approach is possible,^30,31^ and rather fits the framework in Section 3.1 for a continuous outcome or Section 3.4 for a binary outcome.

### 3.4 One-stage meta-analysis using generalised linear mixed models

We now extend the principles above to generalised linear mixed models, for example to derive percentage study weights in a multi-parameter one-stage IPD meta-analysis of binary outcomes. This is especially important when events are rare, as models utilising a more exact (e.g. binomial) within-study likelihood are then preferred.^32^ Further, network meta-analysis of binary outcomes typically uses a one-stage approach.^33^

#### 3.4.1 Model specification

The generalised linear mixed model can be expressed as,^23^

(13) $$\begin{array}{c} Y=\text{E}[Y]+e\\ \text{where g}(\text{E}[Y])=X\beta +\mathrm{Zu} \end{array}$$

and **Y** is the vector of patient responses, **β** is a column vector containing all the unknown fixed parameter effects in the model, **X** and **Z** the design matrices, E[Y] is the expected value of **Y** (conditional on **X**, **Z** and **u**), **u** contains the study-level random effects and **e** contains the participant-level residuals. As in the linear mixed model (8), **u** are typically assumed to follow a multivariate normal distribution with mean vector **0** and variance matrix **G.** We can write V=var(Y)=var(E[Y])+R, where **R** is the residual variance matrix, var(**e**). However, unlike the linear mixed model, **V** is not easily specified; the random effects and residuals are on different scales, and therefore **V** is no longer a simple linear addition of the residual variance matrix plus random effects variance matrix. Brown and Prescott note that a first-order approximation to **V** is,^23^

(14) $$\begin{array}{c} V\approx {\mathrm{BZGZ}}^{T}B+R \end{array}$$

where **B** is a matrix of variance terms, relating to the underlying distribution of data **Y**. For example, for independent responses from the Bernoulli and binomial distributions, **B** would be a diagonal matrix with diagonal entries of $p_{\mathrm{ij}}(1-p_{\mathrm{ij}})$, where post-estimation $p_{\mathrm{ij}}$ would be replaced by p^ij, the best linear unbiased predictor (BLUP) of $Y_{\mathrm{ij}}$ from the model (otherwise known as the empirical Bayes estimates). These are usually available post-estimation in statistical software. For independent responses from a Poisson distribution, the diagonal terms in **B** would simply be the number of predicted events (counts). For further explanation about **V**, we refer to supplementary material 1(b). If residuals are uncorrelated then **R = **AB,^23^ where **A** is typically a matrix of numerical constants that again depends on the distribution of the data. For Bernoulli data and Poisson data with no offset term, **A** is the identity matrix. For binomial data, **A** is a diagonal matrix with diagonal entries $1/n_{\mathrm{ij}}$, where $n_{\mathrm{ij}}$ is the total number of attempts.

#### 3.4.2 Decomposing Fisher’s information matrix

To acquire a decomposition of Fisher’s observed information matrix for β^, we propose using the solution for Itotal(β^) based on maximising the pseudo-likelihood for a linearised pseudo-variable, Y*, as proposed by Wolfinger and O-Connell.^34^ This transforms the response variable **Y** to an approximately linear scale, Y*, and allows the generalised least squares solution for Itotal(β^) to be used. Therefore, our use of the pseudo-likelihood approach is a convenient way to provide a tractable solution for Itotal(β^) in order to decompose it using Itotal(β^)=∑i=1KIi(β^), and then proceed to derive percentage study weights as outlined for general linear mixed models. The process is as follows.

First, the user should calculate var(β^) based on the pseudo-likelihood estimation solution, which is given by Brown and Prescott as,^23^

(15) var(β^)=(Itotal(β^))-1=(XT(VY*)-1X)-1

where

(16) $$\begin{array}{c} V_{Y*}=\text{var}(Y*)={\mathrm{ZGZ}}^{T}+B^{-1}{\mathrm{RB}}^{-1} \end{array}$$

and all matrices are as defined previously. The elements of **G**, **B** and **R** are forced to have their estimated values from the full meta-analysis, as obtained from the model estimation of choice (for example, Gauss-Hermite quadrature). For **B**, this requires the user to specify (functions of) BLUP values (such as p^ij(1-p^ij) for Bernoulli or binomial responses).

As Itotal(β^)=XT(VY*)-1X, the Ii(β^) can be obtained using,

(17a) Ii(β^)=(XiTVY*i-1Xi) where VY*i=ZiGiZiT+Bi-1RiBi-1

and ‘*i*’ again refers to the matrix as specified in the full analysis, except with the removal of participants except those in study *i*. As previously noted for equation (10b), a potentially simpler way of deriving Ii(β^) is to compute

(17b) Ii(β^)=(XTV∇iY*-1X)-1

where **X** is as specified in the full analysis, and $V_{\nabla iY*}$ is the same as $V_{Y*}$ in the full analysis, except that all those participants external to study *i* have corresponding diagonal elements of **V** replaced with a very large number (e.g. 1000000000), and all covariance terms set to zero. This data augmentation approach ensures that all participants, except those in study *i*, have negligible contribution.

#### 3.4.4 Deriving percentage study weights

By obtaining var(β^) and Ii(β^) using equations (15) and (17a/17b), respectively, we can now use equation (11) to derive a weight matrix (Wi(β^)) for each study, and then derive percentage study weights for each parameter using equation (12). An example is given in Section 4.3 for network meta-analysis.

### 3.5 Study information is not only based on the number of participants

Kontopantelis and Reeves^35^ developed a Stata module for generating forest plots from a one-stage, multi-parameter IPD meta-analysis, and state that ‘Patient weights are uniform and therefore each study’s weight is the ratio of its participants over the total number of participants across all studies’. However, this will not usually be correct as the contribution of each study toward Fisher’s total information of a parameter estimate depends on their patients’ elements within $(X^{T}V^{-1}X)$, and these are not necessarily the same. For example, in a one-stage ANCOVA meta-analysis model (see Section 4.1 below), a study’s contribution to **V** will depend on the proportion of patients in the treatment group and its residual variance ($\sigma_{i}^{2}$). Even if all studies were of the same total sample size, those studies with large residual variances and a small proportion of treated patients are likely to have less information relative to other studies with small residual variances and an equal proportion of treated and control patients. This is illustrated further in Section 4.1.

## 4 Applied examples

We now illustrate the proposed methods with some examples.

### 4.1 IPD meta-analysis models to estimate a summary treatment effect for a continuous outcome

Wang et al.^36^ investigated whether active anti-hypertensive treatments lower systolic blood pressure (SBP) compared to placebo or no treatment. Ten trials were ultimately included, providing IPD for a total of 28,581 patients as detailed elsewhere.^15,20^ Table 1 provides the treatment effects in each trial for SBP at the end of follow-up, with the treatment effect defined as the mean difference in treated and control groups after adjusting for baseline SBP. Of interest is using meta-analysis to give a summary treatment effect.

**Table 1.** Study information and percentage study weights toward the overall treatment effect from a one- and two-stage IPD random effects meta-analyses of 10 hypertension trials.

	Number of patients	Final SBP (mm Hg)		Treatment effect estimates in each trial	Weight values used to derive percentage study weights		Percentage study weights		
Trial ID (*i*)	Control/ treatment	Control mean (sd)	Treatment mean (sd)	Treatment effect estimate^a^ θ^i, for each trial from first stage of two-stage IPD meta-analysis (var(θ^i))	Two-stage model *W_i_*	One-stage model (equation (18)) Wi(β^)21,21	Two-stage model: Weights derived using equation (12)^b^	One-stage model (equation (18)): Weights derived using equation (12)	One-stage model (equation (18)): Weights derived using number of patients^c^
1	750/780	139.75 (17.81)	132.85 (16.68)	−6.66 (0.72)	0.095	0.096	11.05	11.06	5.35
2	199/150	179.89 (22.15)	165.06 (20.03)	−14.17 (4.73)	0.064	0.064	7.34	7.32	1.22
3	82/90	170.45 (26.91)	156.88 (21.26)	−12.88 (10.31)	0.043	0.043	4.99	4.99	0.60
4	2371/2427	138.54 (21.26)	130.09 (19.25)	−8.71 (0.30)	0.101	0.102	11.69	11.70	16.79
5	3445/3546	144.25 (17.58)	135.49 (16.32)	−8.70 (0.14)	0.103	0.104	11.93	11.95	24.46
6	1337/1314	164.58 (19.71)	153.99 (20.13)	−10.60 (0.58)	0.097	0.098	11.25	11.25	9.28
7	2371/2365	156.24 (20.12)	145.10 (19.05)	−11.36 (0.30)	0.101	0.102	11.68	11.70	16.57
8	131/137	189.11 (21.90)	171.46 (19.29)	−17.93 (5.82)	0.058	0.058	6.72	6.68	0.94
9	1139/1252	156.55 (16.86)	150.20 (15.84)	−6.55 (0.41)	0.099	0.100	11.50	11.52	8.37
10	2297/2398	165.24 (16.33)	154.87 (16.31)	−10.26 (0.20)	0.102	0.103	11.84	11.86	16.43
					Sum = var(θ^) = 0.87	Sum = var(θ^) = 0.87	100	100	100

The estimated between-study variance (τ^2) from REML was 7.13 in the one-stage analysis, and 7.18 in the two-stage analysis.

a Treatment effect is the mean difference in final systolic blood pressure, after adjusting for baseline (i.e. using an ANCOVA model).

b Equation (12) is equivalent to equation (7) here.

c Kontopantelis and Reeves approach; *θ* is the summary treatment effect.

**Figure 1.**
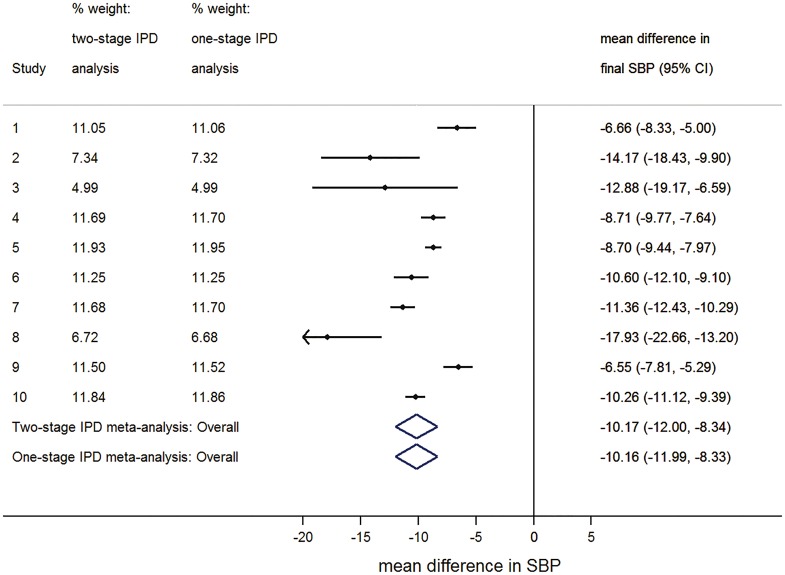
Forest plot for the hypertension meta-analysis comparing the percentage study weights and summary treatment effect results for the two- and one-stage IPD meta-analyses estimated using REML*. *τ^2 was 7.13 in the one-stage analysis and 7.18 in the two-stage analysis.

Figure 1 and Table 1 show the study-specific estimates, the summary meta-analysis results and the percentage study weights from a one- and a two-stage IPD meta-analysis. The two-stage approach used an ANCOVA model (1) in each study separately to obtain treatment effect estimates, which were then pooled using random effects model (3). The alternative one-stage approach also used an ANCOVA model with separate intercepts ($\varphi_{i}$) and baseline adjustments ($\beta_{i}$) per trial and a random treatment effect:

(18) $$\begin{array}{c} y_{\text{F}\mathrm{ij}}=\varphi_{i}+\lambda_{i}y_{\text{B}\mathrm{ij}}+\theta_{i}x_{\mathrm{ij}}+e_{\mathrm{ij}}\\ \theta_{i}=\theta +u_{i}\\ u_{i}∼N(0,\tau^{2})\\ e_{\mathrm{ij}}∼N(0,\sigma_{i}^{2}) \end{array}$$

Parameters for model (18) are as defined previously; the supplementary material 1(a) also gives the matrix expression for the model. The key parameter of interest is the summary treatment effect, θ, defined as the mean difference in SBP at follow-up for the treatment minus the control group, after adjusting for baseline values.

A step-by-step guide to the analyses is given in Appendix 1, alongside SAS Proc Mixed code. REML was used to estimate both models. After fitting each model, var(β^) was obtained directly from the software package, and then Ii(β^) was derived using equation (10b) with variance components held fixed to those in the full meta-analysis. A weight matrix Wi(β^) was then derived for each study using equation (11), and percentage study weights derived using equation (12).

The summary treatment effect estimate and 95% CI are almost identical for the one- and two-stage models (Figure 1). This is unsurprising as all trials are large, and one- and two-stage approaches are considered to be very similar in such situations.^7^ This is reinforced further by examining the percentage study weights (Figure 1, Table 1). These too are almost identical, reflecting that the studies have the same contribution regardless of whether a one- or two-stage approach is used.

In Table 1, we also present percentage study weights based on the proportion of participants, as suggested by Kontopantelis and Reeves.^35^ Clearly, these are somewhat different to those percentage values from the one- and two-stage models, as they ignore differences in residual variances and proportions of participants in the treatment group. For example, for study 3, the contribution based on Kontopantelis and Reeves is 0.60%, whilst in the one- and two-stage analyses, it is 4.99%.

### 4.2 IPD and meta-regression models to evaluate a treatment-covariate interaction

We now extend the hypertension example to compare percentage study weights in three IPD meta-analysis models that are commonly used for estimating treatment-covariate interactions. Interest is in whether age is a treatment effect modifier, and thus whether older patients are more (or less) likely to respond well to hypertension treatment than younger patients.

Let $z_{\mathrm{ij}}$ define the age of patient *j* in study *i*, and let ${\bar{z}}_{i}$ be the mean age of all patients in study *i*. To estimate treatment-covariate interactions, an often used two-stage approach (which is also possible without IPD) is to fit model (1) in the first stage and then a meta-regression in the second stage such as:

(19) θ^i=θi+ɛiθi=θ+γAz¯i+uiui∼N(0,τ2)ɛi∼N(0,var(θ^i))

This performs a weighted regression of the overall treatment effect estimates (θ^i) against the mean age in each study, with the across-study (‘A’) interaction of $\gamma_{\text{A}}$ denoting how a one-unit in mean age increases (or decreases) the overall treatment effect in a study.

A better approach is a two-stage analysis of within-study (‘W’) interactions.^6,15^ Here, model (1) is extended to include an adjustment term for age and an interaction term between age and treatment effect ($\gamma_{\text{W}i}z_{\mathrm{ij}}x_{\mathrm{ij}}$). This model is estimated in each study separately to give γ^Wi and var(γ^Wi), which are pooled, for example in a fixed effect meta-analysis akin to model (2):

(20) γ^Wi=γW+ɛiɛi∼N(0,var(γ^Wi))

This leads to the summary within-study interaction, γ^W, which estimates the change in treatment effect for a one-unit increase in age.

Some researchers suggest one-stage models that amalgamate within-trial and across-trial interactions.^37,38^ This extends model (18) by including an adjustment term for age and an interaction term between age and treatment effect ($\gamma_{\text{WA}}z_{\mathrm{ij}}x_{\mathrm{ij}}$), as follows:

(21) $$\begin{array}{c} y_{\text{F}\mathrm{ij}}=\varphi_{i}+\lambda_{i}y_{\text{B}\mathrm{ij}}+\delta_{i}z_{\mathrm{ij}}+\theta_{i}x_{\mathrm{ij}}+\gamma_{\text{WA}}z_{\mathrm{ij}}x_{\mathrm{ij}}+e_{\mathrm{ij}}\\ \theta_{i}=\theta +u_{i}\\ u_{i}∼N(0,\tau^{2})\\ e_{\mathrm{ij}}∼N(0,\sigma_{i}^{2}) \end{array}$$

This leads to γ^WA, the amalgamated interaction estimate (a weighted average of both within-trial and across-trial associations). This may be prone to ecological bias: that is, the difference between the summary within-trial interaction and the across-trial interaction may be non-zero. The ecological bias can be estimated by re-parameterising as follows,^39^

(22) $$\begin{array}{c} y_{\text{F}\mathrm{ij}}=\varphi_{i}+\lambda_{i}y_{\text{B}\mathrm{ij}}+\delta_{i}z_{\mathrm{ij}}+\theta_{i}x_{\mathrm{ij}}+\gamma_{\text{W}}z_{\mathrm{ij}}x_{\mathrm{ij}}+e_{\mathrm{ij}}\\ \theta_{i}=\theta +\gamma_{\text{E}}{\bar{z}}_{i}+u_{i}\\ u_{i}∼N(0,\tau^{2})\\ e_{\mathrm{ij}}∼N(0,\sigma_{i}^{2}) \end{array}$$

where $\gamma_{\text{E}}$ is the ecological bias ($\gamma_{W}$-$\gamma_{\text{A}}$) and other terms are as defined previously.

REML was used to fit each of the four models, and then percentage weights were obtained in the same manner as described in Appendix 1. The summary results and percentage study weights are very different for γ^A, γ^WA and γ^W (Table 2). The γ^A from meta-regression model (19) is the largest and statistically significant at the 5% level. Model (21) also produces a significant γ^WA, but the γ^W from model (20) is closer to zero and non-significant. The differences arise due to the discrepant use of within-trial and across-trial associations in each analysis, as mentioned. These leads to differences in the percentage study weights for each of γ^A, γ^WA and γ^W. For example, study 1 has a 21.3% contribution toward γ^A in the meta-regression, a 8.9% contribution toward γ^WA in model (21), and an even lower 4.5% contribution toward γ^W in model (20).

**Table 2.** Percentage study weights for the examination of a treatment-age interaction in one- and two-stage IPD random effects meta-analyses of 10 randomised trials of anti-hypertensive treatment versus control on systolic blood pressure.

		Percentage study weights using equation (12)			
Trial ID (*i*)	Mean age (${\bar{z}}_{i}$)	Model (19): Meta-regression to estimate $\gamma_{\text{A}}$ (across-trial interaction)	Model (20): Two-stage approach to estimate $\gamma_{W}$ (within-trial interaction)	Model (21): One-stage approach to estimate $\gamma_{\text{WA}}$ (amalgamated interaction)	Model (22): One-stage approach to estimate $\gamma_{\text{E}}$ (ecological bias)
1	42.27	21.26	4.52	8.88	17.33
2	69.63	2.91	0.66	1.24	2.36
3	73.34	3.64	0.81	1.56	3.13
4	41.56	24.90	11.64	15.13	21.72
5	45.27	16.68	28.78	25.62	19.63
6	70.42	6.15	1.45	2.70	4.93
7	71.59	8.16	17.13	14.81	10.21
8	75.95	7.56	0.29	2.15	5.87
9	66.58	2.35	8.35	6.79	3.74
10	70.23	6.40	26.36	21.11	11.09
Meta-analysis result (95% CI)		γ^A = −0.132 (−0.246, −0.018)	γ^W = −0.050 (−0.116, 0.016)	γ^WA = −0.072 (−0.128, −0.0149)	γ^E = −0.086 (−0.220, 0.048)

Using model (22) to quantify the ecological bias gives γ^E = −0.086, highlighting that the across-trial interaction estimate is more negative in magnitude than the within-trial interaction. The percentage study weights (Table 2) show that studies 1, 4 and 5 have the most contribution toward this bias with weights of 17.3%, 21.7% and 19.6%, respectively. These studies have substantially lower mean ages (close to 40 years) compared to other studies (close to 70 years) and were also the most dominant in the meta-regression.

### 4.3 One- and two-stage network (multivariate) meta-analysis of multiple treatments

Our final example is a network meta-analysis of 28 trials to compare eight thrombolytic treatments after acute myocardial infarction^30^; for brevity, we refer to these treatments as A to H. Of interest is a comparison of the odds of mortality by 30–35 days for each pair of treatments. Table 3 provides the raw data in terms of *r_ij_* (the number of deaths) and *n_ij_* (the total patients) for each treatment group, in each study. It is also provided as a Stata dataset in supplementary material 2. As there are eight treatments, there are 28 comparisons of interest overall; however, only 13 of these comparisons are directly reported in at least one trial. Further, the maximum number of trials providing direct evidence for a particular comparison is only eight (C versus A). Thus, there is potentially large opportunity to borrow strength in this example; in other words, each treatment comparison can utilise correlated and indirect information from other pairs of treatments (contrasts) in the network, alongside any direct evidence.

**Table 3.** Raw data in terms of r (no. of events) and n (total patients) for eight treatment groups (A-H) in the thrombolytic network meta-analysis.

Study	Treatments evaluated (design)^a^	rA	nA	rB	nB	rC	nC	rD	nD	rE	nE	rF	nF	rG	nG	rH	nH
1	A B D	1472	20173	652	10344			723	10328								
2	A C H	1455	13780			1418	13746									1448	13773
3	A C	9	130			6	123										
4	A C	5	63			2	59										
5	A C	3	65			3	64										
6	A C	887	10396			929	10372										
7	A C	7	85			4	86										
8	A C	12	147			7	143										
9	A C	10	135			5	135										
10	A D	4	107					6	109								
11	A F	285	2992									270	2994				
12	A G	10	203											7	198		
13	A H	3	58													2	52
14	A H	3	86													6	89
15	A H	3	58													2	58
16	A H	13	182													11	188
17	B E			522	8488					523	8461						
18	B F			356	4921							757	10138				
19	B F			13	155							7	169				
20	B G			2	26									7	54		
21	B G			12	268									16	350		
22	B H			5	210											17	211
23	B H			3	138											13	147
24	C G					8	132							4	66		
25	C G					10	164							6	166		
26	C G					6	124							5	121		
27	C H					13	164									10	161
28	C H					7	93									5	90

a A = SK; B = AtPA; C = t − PA; D = SK + tPA; E = Ten; F = Ret; G = UK; H = ASPAC as referred to in Lu and Ades.^30^

There are many possible models to perform a network meta-analysis of this data. In particular, either a two-stage ‘contrast-based’ approach, or a one-stage ‘arm-based’ approach can be used, as defined by Salanti et al.^33^ For the two-stage approach, first the data (such as in Table 3) are used to calculate log odds ratio estimates, and their within-study variances and correlations, for each pair of treatments in each study. Second, a multivariate meta-analysis model can be written to synthesise all the effect estimates jointly, whilst accounting for their within and between-study correlations, which enables the incorporation of both direct and indirect evidence toward each summary treatment effect. A common reference group is needed and a design matrix used to express all available treatment comparison estimates (contrasts) in relation to this reference group. For example, with treatment A as the reference group, the log odds ratio estimate for C versus B can be expressed in terms of the log odds ratio estimate for C versus A and the log odds ratio for B versus A (as logOR_(C,B)_ = logOR_(C,A)_ − logOR_(B,A)_). Under an assumption of consistency in the direct and indirect evidence, the general form of this network meta-analysis model can be written as a linear mixed model,^18,29^

(23) θ^i∼N(Xiβ,Si+G)

where θ^i is a vector of treatment effect estimates from study *i*, which has ‘known’ within-study variance matrix $S_{i}$; β is a column vector containing the basic parameters, which are the average treatment effects (for each treatment compared to the chosen reference treatment); $X_{i}$ is a design matrix linking the treatment effect estimates in study *i* to the basic parameters; and **G** contains the variances and covariance of the random effects. Typically, **G** contains diagonal entries of $\tau^{2}$ and off-diagonal entries of 0.5$\tau^{2}$, which ensures there is a common between-study variance of $\tau^{2}$ for all treatment contrasts in the network. Missing treatment effect estimates in some studies can be accommodated easily, for example using data augmentation, and for further details on this model specification, we refer to White et al.^18,29^ Our key focus in this article is that equation (23) is a linear mixed model, and so percentage weights follow according to the description in Section 3.1. For the thrombolytics example, Stata code is given in supplementary material 3 for this model, and β is a 7 by 1 column vector and **G** a 7 by 7 matrix, as there are eight treatments and thus seven basic parameters. The two-stage approach assumes effects follow a multivariate normal distribution both within and between studies. This is an approximation, and a more exact approach is to model the raw data in Table 3 directly in a one-stage, arm-based model that includes a binomial likelihood within studies. These data are essentially the IPD here (as no patient-level covariates are of interest), and so this can be viewed as a one-stage IPD model that falls within the generalised linear mixed model framework outlined in Section 3.4. The general specification of this model can be written as

$$\begin{array}{c} r_{\mathrm{ij}}∼\mathrm{Binomial}(n_{\mathrm{ij}},p_{\mathrm{ij}})\\ \mathrm{logit}(p_{\mathrm{ij}})=\alpha_{i}+X_{i}\beta_{i}\\ \beta_{i}∼N(\beta ,G) \end{array}$$

where $p_{\mathrm{ij}}$ is the probability of death for patients in treatment group *j* of study *i*; $n_{\mathrm{ij}}$ and $r_{\mathrm{ij}}$ are the number of participants and events, respectively, in study *i* for treatment group *j*; the $\alpha_{i}$ are separate study intercept terms, which relate to a chosen reference group in that study; $\beta_{i}$ are the study-specific true treatment effects (in relation to the reference group); and $X_{i}$, β and **G** are as defined above. For the thrombolytics example, this one-stage model can be written as,

(24) $$\begin{array}{c} r_{\mathrm{ij}}∼\mathrm{Binomial}(n_{\mathrm{ij}},p_{\mathrm{ij}})\\ \mathrm{logit}(p_{\mathrm{ij}})=\alpha_{i}+\beta_{1i}{\mathrm{BA}}_{\mathrm{ij}}+\beta_{2i}{\mathrm{CA}}_{\mathrm{ij}}+\beta_{3i}{\mathrm{DA}}_{\mathrm{ij}}+\beta_{4i}{\mathrm{EA}}_{\mathrm{ij}}+\beta_{5i}{\mathrm{FA}}_{\mathrm{ij}}+\beta_{6i}{\mathrm{GA}}_{\mathrm{ij}}+\beta_{7i}{\mathrm{HA}}_{\mathrm{ij}}\\ (\begin{array}{c} \beta_{1i}\\ :\\ \beta_{7i} \end{array})∼N(\beta ,G) \end{array}$$

where $\alpha_{i}$ are separate study intercept terms that relate to the reference group in that study; the *BA_ij_* to *HA_ij_* terms are either 1, 0 or −1 depending on the treatment group that corresponds to $r_{\mathrm{ij}}$; β is a 7 by 1 column vector containing the basic parameters, which are the seven summary treatment effects (for B compared to A, up to H compared to A); and **G** is a 7 by 7 matrix with diagonal entries of $\tau^{2}$ and off-diagonal entries of 0.5$\tau^{2}$. The model is explained in more detail elsewhere,^18,31^ but supplementary material 3 provides SAS code to fit the model and details the coding of the *BA_ij_* to *HA_ij_* variables more explicitly, plus the subsequent derivation of percentage study weights.

The seven summary treatment effect results are given in Table 4 for both the one- (24) and the two-stage (multivariate) approach, with corresponding percentage study weights. The estimate of $\tau^{2}$ was held at the same value in both models, to illustrate a comparison of summary estimates and percentage weights for one- and two-stage network meta-analyses when τ^2 was the same. We chose a τ^2 of 0.00023, which was the REML estimate from the two-stage approach. The ML estimate from the one-stage approach was zero and thus only slightly smaller. The summary estimates and percentage study weights barely changed when using τ^2 = 0, but for completeness, these are shown in supplementary material 4.

**Table 4.** Percentage study weights (using equation (12)) and summary treatment effects for one- and two-stage network meta-analysis models assuming consistency.^a^

	Percentage weights from the two-stage network (multivariate) meta-analysis							Percentage weights from the one-stage network meta-analysis						
	B vs A	C vs A	D vs A	E vs A	F vs A	G vs A	H vs A	B vs A	C vs A	D vs A	E vs A	F vs A	G vs A	H vs A
Study 1	81.14	0.01	97.70	26.67	18.44	0.53	0.01	80.62	0.01	97.65	26.44	18.24	0.56	0.03
Study 2	0.02	58.05	0	0.01	0	0.23	90.34	0.03	57.88	0	0.01	0.01	0.22	89.78
Study 3	0	0.35	0	0	0	0	0.06	0	0.36	0	0	0	0	0.06
Study 4	0	0.14	0	0	0	0	0.02	0	0.17	0	0	0	0	0.03
Study 5	0	0.15	0	0	0	0	0.02	0	0.15	0	0	0	0	0.02
Study 6	0	39.18	0	0	0	0.15	6.33	0.01	39.14	0	0	0	0.15	6.39
Study 7	0	0.25	0	0	0	0	0.04	0	0.27	0	0	0	0	0.04
Study 8	0	0.43	0	0	0	0	0.07	0	0.46	0	0	0	0	0.07
Study 9	0	0.33	0	0	0	0	0.05	0	0.37	0	0	0	0	0.06
Study 10	0.05	0	0.55	0.02	0.01	0	0	0.05	0	0.57	0.02	0.01	0	0
Study 11	10.58	0	0.99	3.48	46.54	0.07	0	10.52	0	0.97	3.45	46.50	0.07	0
Study 12	0.13	0.07	0.01	0.04	0.03	19.36	0.01	0.13	0.07	0.01	0.04	0.03	19.28	0.01
Study 13	0	0.03	0	0	0	0	0.18	0	0.03	0	0	0	0	0.18
Study 14	0	0.05	0	0	0	0	0.29	0	0.05	0	0	0	0	0.33
Study 15	0	0.03	0	0	0	0	0.18	0	0.03	0	0	0	0	0.18
Study 16	0	0.14	0	0	0	0	0.86	0	0.14	0	0	0	0	0.86
Study 17	0	0	0	67.13	0	0	0	0	0	0	67.21	0	0	0
Study 18	5.82	0	0.54	1.91	33.80	0.04	0	5.78	0	0.53	1.90	33.86	0.04	0
Study 19	0.12	0	0.01	0.04	0.68	0	0	0.13	0	0.01	0.04	0.74	0	0
Study 20	0.11	0.03	0.01	0.04	0.03	6.72	0	0.13	0.03	0.01	0.04	0.03	8.17	0
Study 21	0.51	0.12	0.05	0.17	0.12	31.02	0.02	0.50	0.11	0.05	0.16	0.11	30.45	0.02
Study 22	0.77	0.09	0.07	0.25	0.17	0	0.56	1.05	0.12	0.10	0.35	0.24	0	0.76
Study 23	0.49	0.06	0.05	0.16	0.11	0	0.35	0.76	0.09	0.07	0.25	0.17	0	0.55
Study 24	0.08	0.09	0.01	0.03	0.02	12.07	0.01	0.08	0.08	0.01	0.02	0.02	11.04	0.01
Study 25	0.11	0.12	0.01	0.04	0.03	17.24	0.02	0.12	0.13	0.01	0.04	0.03	17.77	0.02
Study 26	0.08	0.09	0.01	0.03	0.02	12.55	0.01	0.08	0.09	0.01	0.03	0.02	12.23	0.01
Study 27	0	0.14	0	0	0	0	0.36	0	0.14	0	0	0	0	0.37
Study 28	0	0.07	0	0	0	0	0.19	0	0.07	0	0	0	0	0.19
Total	100	100	100	100	100	100	100	100	100	100	100	100	100	100
Summary log odds ratio (s.e.)	−0.161 (0.046)	0.002 (0.032)	−0.044 (0.049)	−0.156 (0.080)	−0.113 (0.062)	−0.197 (0.222)	0.014 (0.039)	−0.166 (0.046)	0.003 (0.032)	−0.045 (0.049)	−0.160 (0.080)	−0.117 (0.062)	−0.197 (0.219)	0.017 (0.039)

a The between-study variance was estimated at 0.000231 for the two-stage analysis, and kept at this value for the one-stage analysis; this was done to illustrate a comparison of the summary results and percentage weights for one- and two-stage models when the between-study variance was the same. Results for different between-study variance estimates are given in supplementary material 4.

Table 4 shows that the summary effects and percentage study weights are almost identical for both one- and two-stage models. Thus, the multivariate normal approximation to the one-stage binomial approach performs well, and the contribution of each study is barely changed. In other network meta-analysis applications, this may not be the case, especially when there are rare events as then the one-stage approach is preferred.^40^

The described one- and two-stage models are known as ‘consistency’ models as they assume direct and indirect evidence are consistent (in agreement). One can extend them to add inconsistency terms for each treatment contrast, either by including them as additional random effects with mean zero,^41–43^ or by including them as fixed effects.^18,31^ When the latter is done for this dataset, as described by White et al., there is generally strong support for the consistency model, as seven of the eight inconsistency parameters are smaller than their standard errors. The only inconsistency parameter that is statistically significant (p = 0.024) relates to the indirect evidence toward the H versus B treatment comparison. Though this may be a chance finding, it is useful to examine it. Derivation of percentage study weights is helpful here to reveal which studies are contributing most toward the apparent inconsistency. Just four studies contribute: study 1 (17.1%), study 2 (16.8%), study 22 (39.6%) and study 23 (26.5%). Studies 22 and 23 have about two thirds of the weight, and they both compare H and B. They represent the direct evidence, which is discrepant from the indirect evidence coming from studies 1 and 2, which compare A, B and D, and A, C and H, respectively. Therefore, the inconsistency is arising from the indirect comparison through A (i.e. the loop involving A, B and H), and our percentage weights reveal that studies 1 and 2 have a very similar contribution toward this indirect evidence. In situations like this, it is helpful for analysts to compare studies that give indirect information with those that give direct information, to see if there are any obvious differences that might explain the inconsistency. Nothing was identified here and, as mentioned above, there is generally little evidence to support inconsistency in this network meta-analysis overall.

## 5 Discussion

**Figure 2.**
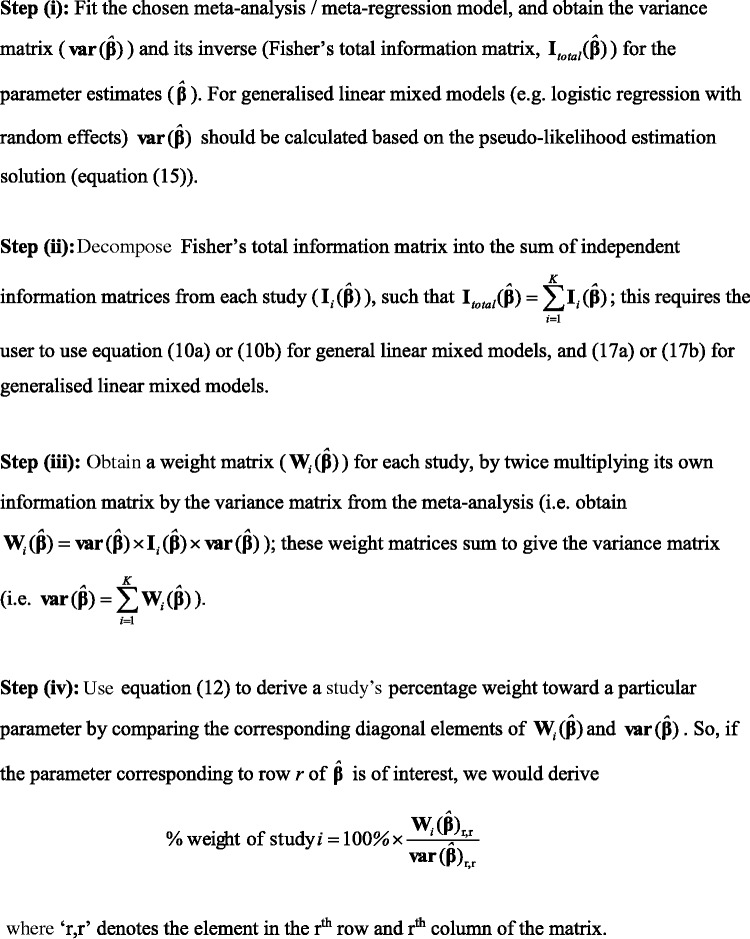
Step-by-step guide to the derivation of percentage study weights in meta-analysis and meta-regression models.

In this article, we proposed how to calculate percentage study weights in multi-parameter meta-analysis and meta-regression models. Figure 2 summarises the four necessary steps. The approach generalises how percentage weights are calculated in a traditional single parameter meta-analysis, and now allows percentage weights to be derived for more complex models including meta-regression, one-stage IPD analyses and multivariate and network meta-analysis. Though focus will usually be on deriving percentage study weights toward summary (treatment) effects and (treatment-covariate) interactions, our approach is applicable for any parameter that is specified within a meta-analysis model that can be expressed as a general or generalised linear mixed model. This was shown in our second and third examples, where percentage study weights toward bias and inconsistency terms were examined, which may be of more interest to methodologists than clinicians.

Our proposal assumes that studies are independent, and utilises a decomposition of Fisher’s observed information matrix to decompose the total variance matrix of parameter estimates into study-specific contributions, from which percentage weights are derived. The variance scale is a sensible one for quantifying percentage weights, as the variance of a parameter estimate summarises the total information data toward that estimate, which comprises both direct (from the parameter itself) and indirect information (from other, correlated parameters in the model). Derivation of percentage study weights is simple for traditional (single parameter) meta-analysis models, and is straightforward to extend for one-stage (multiple parameter) general linear mixed models as the information matrix is not dependent on the parameter estimates themselves; thus the study-specific information matrix (Ii(β^)) can easily be derived by using any software that can re-fit the one-stage model including just study *i*, whilst allowing variance terms to be fixed. However, for generalised linear models, the residual variances are a function of the expected response values, and therefore it is harder to re-fit models whilst fixing variances after taking out studies (or cases^44,45^). Supplementary material 1 illustrates this with an example. Furthermore, the original scale of the analysis is non-linear and so identifying sums of information matrices is not immediate. For this reason, we proposed utilising the pseudo-likelihood approach of Wolfinger and O-Connell,^34^ which transforms the response data to a pseudo-linearised variable, so that the maximum likelihood (generalised least squares) solution for Fisher’s information matrix can again be utilised and decomposed.

In many situations, percentage study weights derived from a two-stage approach will be a close approximation to those from a one-stage approach, as two- and one-stage analyses often give similar meta-analysis results, as seen in examples 1 and 3.^8^ However, in our second example, there were substantial clinically and statistically important differences in the one- and two-stage models, as they were making discrepant use of between-study information. Similarly, when there are rare outcomes, then the percentage study weights for one- and two-stage models may differ, as the former uses a more exact (e.g. binomial) within-study likelihood and so summary results often differ.^32,40^ Recent evidence suggests one-stage models are increasingly being used.^2^

For time-to-event data, it has been shown how the Cox regression model can be fitted using a Poisson model due to the shared form of the contribution to the partial log-likelihood, by splitting follow-up time into as many intervals as there are events.^46^ Crowther et al. show how to perform a one-stage fixed or random effects IPD meta-analysis of survival data in this manner,^47^ which additionally provides the baseline hazard, and may be useful for prognostic modelling. Therefore, following this approach, meta-analysts can derive percentage study weights as described above for generalised linear mixed models. However, as each patient now has multiple rows (one for each interval), the approach for model estimation and subsequently derivation of percentage study weights may be computationally intensive. To address this, one can rather use larger intervals, such as quarter year or half year, to approximate the likelihood and improve computation time for derivation of percentage study weights. When analysing without the inclusion of continuous individual-level covariates, it may be possible to collapse the IPD to just a few rows per study to make the computation easier. This approach was used in the network meta-analysis example.

Our work complements the PRISMA-IPD reporting guidelines, which state that: ‘… the display of forest plots for key outcomes is advocated, *irrespective of the type of approach to statistical analysis*’.^48^ Percentage study weights should be routinely presented on forest plots, which in our experience, are rarely provided for IPD meta-analyses. Most software packages link forest plots with a two-stage meta-analysis. To our knowledge, the only exception is the ‘ipdforest’ module in Stata, which provides a forest plot with a one-stage summary result after either a one-stage linear or logistic (mixed) model is fitted. However, this module computes percentage study weights as the ratio of a study’s total participants over the total number of participants across all studies. Our first example (Table 1) showed that this does not correspond closely to percentage values derived using our approach. We therefore hope the ‘ipdforest’ module may be updated in the future.

Our work should also be applicable to other types of datasets involving independent clusters, where the weight of each cluster is of interest. This may include the analysis of a multi-centre randomised trial, or the analysis of an observational study with clustering by hospital, practice or region.

Finally, our described approach utilises Fisher’s information matrix for β^, but ignores Fisher’s information matrix for any estimated variance parameters. In other words, our derivation of percentage study weights ignores uncertainty in variance parameter estimates, and thus assumes that Fisher’s information matrix for β^ is independent from Fisher’s information matrix for estimated (residual and between-study) variances. We consider this is sensible, as the most commonly used meta-analysis models either assume variance estimates are ‘known’ or, *post*-estimation, inflate confidence intervals for β^ to account for uncertainty in variance estimates.^49^ For the latter situation, our approach should be valid as it focuses on decomposing the observed information toward β^ itself, which does not change when post-estimation correction factors (such as Hartung-Knapp and robust variance estimators^50–52^) are applied in order to derive confidence intervals. Nevertheless, further consideration of this issue is needed, and in particular, whether our approach should be modified to situations where the uncertainty in variance estimates is propagated toward the estimation of β^ itself.^53^ A further extension might consider percentage weights toward variance estimates, but this should be considered in a standard (pair-wise) meta-analysis first, before extension to multi-parameter (e.g. network) meta-analysis models.

In summary, we have proposed how to derive percentage study weights for multi-parameter meta-analysis and meta-regression models, which extend those for single parameter models. We hope this encourages researchers to reveal the contribution of each study toward meta-analysis results, and will be useful for methodologists aiming to understand and explain differences or potential biases in meta-analysis models.

## Supplementary Material

Supplementary material

Supplementary material

Supplementary material

Supplementary material

## Appendix 1

### Example SAS Proc Mixed code to obtain percentage study weights toward one-stage IPD meta-analysis model (18)

The method described below is a way of utilising existing software to obtain percentage study weights in a convenient way for an IPD meta-analysis model with a continuous outcome. It avoids the user needing to specify the entire **X** and **V** matrices by hand. It can also be applied to a meta-regression model, as this can also be expressed within the general linear mixed model framework.

**Step (i):** Fit one-stage IPD meta-analysis model (18) to all studies

proc mixed cl method = reml data = hypertension;

class idnr trial;

/* save the estimated variance matrix of the parameter estimates as a dataset ‘varbfull’ */

ods listing;

ods output covb = varbfull;

/* write down the model, where sbpl is final systolic blood pressure, idnr is the unique id number of the patient, trial is the trial identifier, sbpi is the baseline systolic blood pressure, and treat is the treatment group identifier */

model sbpl = trial trial*sbpi treat / noint s cl covb;

/* place a random effect on the treatment effect to allow for between-trial heterogeneity */

random treat / type = un subject = trial;

/* the following ensures a separate residual variance per trial */

repeated / type = un subject = idnr(trial) group = trial;

run;

This provides the summary meta-analysis results, including β^, the residual variances (one per study), the between-study variance, and vartotal(β^)=(XTV-1X)-1, the latter which is saved as ‘varbfull’. There are 21 estimated parameters in the main equation, and parameter 21 is the treatment effect (θ). Therefore, the (21,21) entry of vartotal(β^) = var(θ^) = 0.868.

**Step (ii):** implement equation 10(b) to obtain Ii(β^). For example, for study 1 do the following. Re-fit model (18) to all studies but for studies other than study 1, give the residual variances a large value (to ensure they have negligible contribution); and for study 1, hold fixed residual and between-study variances at those values from the full analysis.

proc mixed cl method = reml data = hypertension;

class idnr trial;

ods listing;

ods output covb = varb;

model sbpl = trial trial*sbpi treat / noint s cl covb;

random treat / type = un subject = trial;

repeated / type = un subject = trial(idnr) group = trial;

/* use the ‘parms’ statement to specify values for the residual variances and between-study variance */

parms

/* set the between-study variance at the value from the full analysis obtained from step (i) */

7.1295

/* fix the residual variance for study 1 at its value from the full analysis obtained from step (i) */

276.31

/* set the residual variances for study 2 to 10 at a large value */

100000000000

100000000000

100000000000

100000000000

100000000000

100000000000

100000000000

100000000000

100000000000

/* use ‘eqcons’ to ensure that SAS holds fixed the 11 specified variances */

/ eqcons = 1 to 11;

run;

We then simply need to invert the stored var(β^) (named ‘varb’) to obtain I1(β^)

**Step (iii):** obtain a weight matrix (Wi(β^)) for each study, by twice multiplying its own information matrix by the variance matrix from the meta-analysis. Below is example code to do the matrix calculations for study 1:

proc iml;

/* convert the stored dataset ‘varb’ from the analysis of study 1 into a matrix /*

use varb;

read all var {Col1 Col2 Col3 Col4 Col5 Col6 Col7 Col8 Col9 Col10 Col11 Col12 Col13Col14 Col15 Col16 Col17 Col18 Col19 Col20 Col21} into varb;

/* invert this matrix to obtain Fisher’s information matrix for study 1 */

fish = inv(varb);

/* convert the stored dataset ‘varbfull’ into a matrix */

use varfull;

read all var {Col1 Col2 Col3 Col4 Col5 Col6 Col7 Col8 Col9 Col10 Col11 Col12 Col13Col14 Col15 Col16 Col17 Col18 Col19 Col20 Col21} into varfull;

/* obtain the weight matrix (W_1_) for study 1 */

weight = varfull*fish*varfull;

quit;

The element (21,21) of W1(β^) is 0.096

**Step (iv):** Apply equation (12) to obtain the percentage weights of interest. For example, the percentage weight of study 1 toward the summary treatment effect estimate is:

%weight of studyi=100%×W1(β^)21,21var(β^)21,21=100%×0.0960.868=11.06%
