# Supplementary material for: Deriving percentage study weights in multi-parameter meta-analysis models: with application to meta-regression, network meta-analysis and one-stage individual participant data models
Source: Stat Methods Med Res. 2017 Feb 6;27(10):2885–905. doi: 10.1177/0962280216688033 (PMC6146321; doi:10.1177/0962280216688033)
Supplement: Supplementary material [file Supplemental_Material_1.pdf]

Supplementary material 1

(a): One-stage analysis of covariance (ANCOVA) model in matrix format

For example, assuming for simplicity there are 3 trials and 2 patients in each trial, a one-stage ANCOVA model could be written:

$$\mathbf{Y} = \mathbf{X\beta} + \mathbf{Zu} + \mathbf{e}$$

$$\begin{pmatrix} y_{F11} \\ y_{F12} \\ y_{F21} \\ y_{F22} \\ y_{F31} \\ y_{F32} \end{pmatrix} = \begin{pmatrix} 1 & 0 & 0 & y_{B11} & 0 & 0 & x_{11} \\ 1 & 0 & 0 & y_{B12} & 0 & 0 & x_{12} \\ 0 & 1 & 0 & 0 & y_{B21} & 0 & x_{21} \\ 0 & 1 & 0 & 0 & y_{B22} & 0 & x_{22} \\ 0 & 0 & 1 & 0 & 0 & y_{B31} & x_{31} \\ 0 & 0 & 1 & 0 & 0 & y_{B32} & x_{32} \end{pmatrix} \begin{pmatrix} \phi_1 \\ \phi_2 \\ \phi_3 \\ \lambda_1 \\ \lambda_2 \\ \lambda_3 \\ \theta \end{pmatrix} + \begin{pmatrix} x_{11} & 0 & 0 & 0 & 0 & 0 \\ 0 & x_{12} & 0 & 0 & 0 & 0 \\ 0 & 0 & x_{21} & 0 & 0 & 0 \\ 0 & 0 & 0 & x_{22} & 0 & 0 \\ 0 & 0 & 0 & 0 & x_{31} & 0 \\ 0 & 0 & 0 & 0 & 0 & x_{32} \end{pmatrix} \begin{pmatrix} u_1 \\ u_1 \\ u_2 \\ u_2 \\ u_3 \\ u_3 \end{pmatrix} + \begin{pmatrix} e_{11} \\ e_{12} \\ e_{21} \\ e_{21} \\ e_{31} \\ e_{32} \end{pmatrix}$$

where $y_{\mathrm{F}ij}$ and $y_{\mathrm{B}ij}$ denote the final and baseline value, respectively, for patient *j* in trial *i* (where *B* indicates baseline), and x_{ij} is 0/1 for participants in the control/treatment group. Further

$$\begin{pmatrix} e_{11} \\ e_{12} \\ e_{21} \\ e_{21} \\ e_{31} \\ e_{32} \end{pmatrix} \sim N(\mathbf{0},\mathbf{R}) \qquad \mathbf{R} = \begin{pmatrix} \sigma_1^2 & 0 & 0 & 0 & 0 & 0 \\ 0 & \sigma_1^2 & 0 & 0 & 0 & 0 \\ 0 & 0 & \sigma_2^2 & 0 & 0 & 0 \\ 0 & 0 & 0 & \sigma_2^2 & 0 & 0 \\ 0 & 0 & 0 & 0 & \sigma_3^2 & 0 \\ 0 & 0 & 0 & 0 & 0 & \sigma_3^2 \end{pmatrix}$$

$$\begin{pmatrix} u_1 \\ u_1 \\ u_2 \\ u_2 \\ u_3 \\ u_3 \end{pmatrix} \sim N(\mathbf{0},\mathbf{G}) \qquad \mathbf{G} = \begin{pmatrix} \tau^2 & 0 & 0 & 0 & 0 & 0 \\ 0 & \tau^2 & 0 & 0 & 0 & 0 \\ 0 & 0 & \tau^2 & 0 & 0 & 0 \\ 0 & 0 & 0 & \tau^2 & 0 & 0 \\ 0 & 0 & 0 & 0 & \tau^2 & 0 \\ 0 & 0 & 0 & 0 & 0 & \tau^2 \end{pmatrix}$$

where e_{ij} are the within-trial residual errors, which are assumed normally distributed with mean zero and residual variance σ_i^2 (that is, a separate residual variance is allowed in each trial), and the u_i are the between-study errors in the true treatment effect, which again are assumed normally distributed with a mean of zero and between-study variance τ^2.

The specification of **V** is:

$$\mathbf{V} = \text{var}(\mathbf{Y}) = \text{var}(\mathbf{X}\boldsymbol{\beta} + \mathbf{Zu} + \mathbf{e}) = \text{var}(\mathbf{Zu} + \mathbf{e}) = \mathbf{Z}\text{var}(\mathbf{u})\mathbf{Z}^T + \text{var}(\mathbf{e}) = \mathbf{ZGZ}^T + \mathbf{R}$$

$$= \begin{pmatrix} x_{11}^2\tau^2 + \sigma_1^2 & 0 & 0 & 0 & 0 & 0 \\ 0 & x_{12}^2\tau^2 + \sigma_1^2 & 0 & 0 & 0 & 0 \\ 0 & 0 & x_{21}^2\tau^2 + \sigma_2^2 & 0 & 0 & 0 \\ 0 & 0 & 0 & x_{22}^2\tau^2 + \sigma_2^2 & 0 & 0 \\ 0 & 0 & 0 & 0 & x_{31}^2\tau^2 + \sigma_3^2 & 0 \\ 0 & 0 & 0 & 0 & 0 & x_{32}^2\tau^2 + \sigma_3^2 \end{pmatrix}$$

The derivation of percentage study information for study i requires the calculation of $\mathbf{I}_i(\hat{\boldsymbol{\beta}})$ using equation (8a) or (8b), with the latter involving refitting the one-stage model but forcing the diagonal components of **V** to be very large (say 1000000000) for study i and held fixed at their values from the full analysis for all other studies. See Appendix A.

(b): Examples of one-stage IPD meta-analysis models in the one-stage generalised linear mixed model framework

A one-stage IPD random effects meta-analysis model can be expressed in the generalised linear mixed model framework. For instance, we may be interested in fitting a one-stage random effects logistic regression for a binary outcome from multiple randomised trials:

$$\log\left(\frac{\mathrm{E}(y_{ij})}{1 - \mathrm{E}(y_{ij})}\right) = \log\left(\frac{p_{ij}}{1 - p_{ij}}\right) = \alpha_i + \theta_i x_{ij}$$
$$\theta_i = \theta + u_i$$
$$u_i \sim N(0, \tau^2)$$

where y_{ij} is 1 or 0 for those with or without the outcome, respectively, p_{ij} is the probability of patient *j* in study *i* experiencing the outcome, and α_i is the log-odds of the outcome in study *i* for the control group, x_{ij} is 1 for those in the treatment group and 0 for control group, and u_i denotes a random effect that indicates the treatment effect (log odds ratio) in the ith trial, θ_i, is assumed normally distributed about a summary (mean) treatment effect, θ, with between-study variance, τ^2. Assuming, for simplicity, there are 3 trials each containing 2 patients, the matrix specification for the above equation is:

$$\mathbf{Y} = \mathrm{E}(\mathbf{Y}) + \mathbf{e}$$

$$\begin{pmatrix} y_{11} \\ y_{12} \\ y_{21} \\ y_{22} \\ y_{31} \\ y_{32} \end{pmatrix} = \mathrm{E}(\mathbf{Y}) + \begin{pmatrix} e_{11} \\ e_{12} \\ e_{21} \\ e_{21} \\ e_{31} \\ e_{32} \end{pmatrix}$$

$$\mathrm{g}(\mathrm{E}(\mathbf{Y})) = \log\left(\mathrm{E}(\mathbf{Y})/(1 - \mathrm{E}(\mathbf{Y}))\right)$$

$$= \mathbf{X\beta} + \mathbf{Zu} = \begin{pmatrix} 1 & 0 & 0 & x_{11} \\ 1 & 0 & 0 & x_{12} \\ 0 & 1 & 0 & x_{21} \\ 0 & 1 & 0 & x_{22} \\ 0 & 0 & 1 & x_{31} \\ 0 & 0 & 1 & x_{32} \end{pmatrix} \begin{pmatrix} \phi_1 \\ \phi_2 \\ \phi_3 \\ \theta \end{pmatrix} + \begin{pmatrix} x_{11} & 0 & 0 & 0 & 0 & 0 \\ 0 & x_{12} & 0 & 0 & 0 & 0 \\ 0 & 0 & x_{21} & 0 & 0 & 0 \\ 0 & 0 & 0 & x_{22} & 0 & 0 \\ 0 & 0 & 0 & 0 & x_{31} & 0 \\ 0 & 0 & 0 & 0 & 0 & x_{32} \end{pmatrix} \begin{pmatrix} u_1 \\ u_1 \\ u_2 \\ u_2 \\ u_3 \\ u_3 \end{pmatrix}$$

As a second example, consider a one-stage random effects Poisson regression for examining rates from multiple randomised trials, where each patient provides the number of outcomes y_{ij} (e.g. number of hospitalisations) over a follow-up period, t_{ij}:

$$\log(\mathrm{E}(y_{ij})) = \log(\mathrm{E}(\lambda_{ij} t_{ij})) = \alpha_i + \theta_{\mathrm{i}} x_{ij} + \log(t_{ij})$$
$$\theta_i = \theta + u_i$$
$$u_i \sim N\left(0, \tau^2\right)$$

Here $\log(t_{ij})$ is the offset term, α_i is the log-rate of the outcome in study *i* for the control group, λ_{ij} is the rate of the outcome for patient *j* in study i, x_{ij} is 1 for those in the treatment group and 0 for control group, and θ denotes the summary log rate ratio (i.e. the treatment effect) in the ith study. Assuming, for simplicity, there are 3 trials each containing 2 patients, the matrix specification for the above equation is:

$$\mathbf{Y} = \mathrm{E}(\mathbf{Y}) + \mathbf{e}$$

$$\begin{pmatrix} y_{11} \\ y_{12} \\ y_{21} \\ y_{22} \\ y_{31} \\ y_{32} \end{pmatrix} = \mathrm{E}(\mathbf{Y}) + \begin{pmatrix} e_{11} \\ e_{12} \\ e_{21} \\ e_{21} \\ e_{31} \\ e_{32} \end{pmatrix}$$

$$\mathrm{g}(\mathrm{E}(\mathbf{Y})) = \log\big(\mathrm{E}(\mathbf{Y})\big)$$

$$= \mathbf{X\beta} + \mathbf{Zu} = \begin{pmatrix} 1 & 0 & 0 & x_{11} & \log(t_{11}) \\ 1 & 0 & 0 & x_{21} & \log(t_{12}) \\ 0 & 1 & 0 & x_{21} & \log(t_{21}) \\ 0 & 1 & 0 & x_{22} & \log(t_{22}) \\ 0 & 0 & 1 & x_{31} & \log(t_{31}) \\ 0 & 0 & 1 & x_{32} & \log(t_{32}) \end{pmatrix} \begin{pmatrix} \phi_1 \\ \phi_2 \\ \phi_3 \\ \theta \\ 1 \end{pmatrix} + \begin{pmatrix} x_{11} & 0 & 0 & 0 & 0 & 0 \\ 0 & x_{12} & 0 & 0 & 0 & 0 \\ 0 & 0 & x_{21} & 0 & 0 & 0 \\ 0 & 0 & 0 & x_{22} & 0 & 0 \\ 0 & 0 & 0 & 0 & x_{31} & 0 \\ 0 & 0 & 0 & 0 & 0 & x_{32} \end{pmatrix} \begin{pmatrix} u_1 \\ u_1 \\ u_2 \\ u_2 \\ u_3 \\ u_3 \end{pmatrix}$$

Recall from the main paper that a first-order approximation to **V** is,[1]

$$\mathbf{V} \approx \mathbf{BZGZ^TB} + \mathbf{R} \qquad (14)$$

where **B** is a matrix of variance terms, relating to the underlying distribution of data **Y**. For example, for independent responses from the Bernoulli and binomial distributions, **B** would be a diagonal matrix with diagonal entries of $p_{ij}(1 - p_{ij})$ where post-estimation p_{ij} would be replaced by $\hat{p}_{ij}$, the best linear unbiased predictor (BLUP) of Y_{ij} from the model (otherwise known as the empirical Bayes estimates). These are usually available post-estimation in statistical software. For independent responses from a Poisson distribution, the diagonal terms in **B** would simply be the number of predicted events (counts).

Brown and Prescott note that $\mathbf{R} = \mathbf{AB}$ if residuals are uncorrelated (that is, patient-level responses are independent in each study),[1] where **B** is as defined above and **A** is typically a matrix of numerical constants that again depends on the distribution of the data. For Bernoulli data and Poisson data with no offset term, then **A** is the identity matrix. For Poisson data (e.g. with the number of events per individual as the response) with an offset term, denoted by offset_{ij} (e.g. length of follow-up), **A** is diagonal with entries $1/\mathit{offset}_{ij}$. For binomial data (for example, with the number of successes per individual as the response), **A** is a diagonal matrix with diagonal entries $1/n_{ij}$ where n_{ij} is the total number of attempts.

If residuals are correlated in the same study (for example, in a repeated measures model[2]) then Brown and Prescott also note that,[1]

$$\mathbf{R} = \mathbf{A}^{1/2}\mathbf{B}^{1/2}\mathbf{P}\mathbf{B}^{1/2}\mathbf{A}^{1/2} \quad (15)$$

where **P** is a correlation matrix defined on the linear scale. For brevity, we do not consider examples with correlated responses or give further details of the theory here, and refer the reader to more in-depth explanations elsewhere.[1, 3, 4]

In terms of the pseudo-likelihood solution of $\mathbf{V} = \mathrm{var}(\mathbf{Y}) \approx \mathrm{var}(\mathrm{g}^{-1}(\mathrm{E}(\mathbf{Y}))) + \mathbf{R} = \mathbf{BZGZ^TB} + \mathbf{A}^{1/2}\mathbf{B}^{1/2}\mathbf{P}\mathbf{B}^{1/2}\mathbf{A}^{1/2}$, for the logistic regression example above with 6 patients, we have that **A** and **P** are 6 by 6 identity matrices, and **B** is a 6 by 6 diagonal matrix with entries of $p_{ij}(1 - p_{ij})$. For the Poisson example above with 6 patients, we have **P** as a 6 by 6 identify matrix, **A** as a 6 by 6 diagonal matrix with entries of $1/t_{ij}$ and **B** as a 6 by 6 diagonal matrix with entries of the number of predicted events, $\hat{\lambda}_{ij}t_{ij}$.

(c): Example to demonstrate why simply removing a study and re-estimating the model is not the correct approach to obtaining percentage study information

To illustrate this, consider a simple example. Assume there are two studies, and the probability of a binary event is of interest. Let there be 10 patients in each study, with 2 events in study one and 8 events in study two. If a simple logistic regression model containing just a fixed intercept term is applied to this data, the estimated probability ($\hat{p}$) of an event is 0.5 with a variance of logit($\hat{p}$) = 0.2; thus Fisher's total information is 1/0.2 = 5. If we repeat the analysis with study two excluded, then we get $\hat{p}$ = 0.2 with variance of logit($\hat{p}$) = 0.625; thus Fisher's information when including just study one is 1/0.625 = 1.6. Similarly, when excluding study one $\hat{p}$ = 0.8 with variance of logit($\hat{p}$) = 0.625, and Fisher's information is again 1.6. Clearly, the two study-specific Fisher's information values do not sum to the total information from the full meta-analysis. This is due to the large differences in the estimated event probability from each analysis, which leads to vast differences in the residual variances for each participant (which are defined by $\hat{p}(1-\hat{p})$). In other words, the analyses excluding a study do not reflect the full analysis, and so the observed information is

not transferable. Our proposed approach addresses this by ensuring residual (and between-study) variances are always fixed at their values from the full analysis.

Reference List

1. Brown H and Prescott R. *Applied Mixed Models in Medicine*. Chichester: John Wiley, 1999.

2. Jones AP, Riley RD, Williamson PR and Whitehead A. Meta-analysis of individual patient data versus aggregate data from longitudinal clinical trials. *Clin Trials*. 2009; 6: 16-27.

3. Stroup WW. *Generalized Linear Mixed Models: Modern Concepts, Methods and Applications*. Florida: CRC Press, 2012.

4. McCulloch CE, Searle SR and Neuhaus JM. *Generalized, Linear, and Mixed Models (2nd Edition)*. New York: Wiley, 2008.
