# Supplementary material for: Deriving percentage study weights in multi-parameter meta-analysis models: with application to meta-regression, network meta-analysis and one-stage individual participant data models
Source: Stat Methods Med Res. 2017 Feb 6;27(10):2885–905. doi: 10.1177/0962280216688033 (PMC6146321; doi:10.1177/0962280216688033)
Supplement: Supplementary material [file Supplemental_Material_4.pdf]

Supplementary material 4: Percentage study weights (using equation (12)) and summary treatment effects for one-stage and two-stage network meta-analysis models assuming consistency*

	**Percentage weights from the two-stage network (multivariate) meta-analysis**							**Percentage weights from the one-stage network meta-analysis**						
	**B vs A**	**C vs A**	**D vs A**	**E vs A**	**F vs A**	**G vs A**	**H vs A**	**B vs A**	**C vs A**	**D vs A**	**E vs A**	**F vs A**	**G vs A**	**H vs A**
**Study 1**	81.14	0.01	97.70	26.67	18.44	0.53	0.01	80.63	0.01	97.65	26.43	18.23	0.56	0.03
**Study 2**	0.02	58.05	0	0.01	0	0.23	90.34	0.03	57.88	0	0.01	0.01	0.22	89.77
**Study 3**	0	0.35	0	0	0	0	0.06	0	0.36	0	0	0	0	0.06
**Study 4**	0	0.14	0	0	0	0	0.02	0	0.17	0	0	0	0	0.03
**Study 5**	0	0.15	0	0	0	0	0.02	0	0.15	0	0	0	0	0.02
**Study 6**	0	39.18	0	0	0	0.15	6.33	0.01	39.14	0	0	0	0.15	6.41
**Study 7**	0	0.25	0	0	0	0	0.04	0	0.27	0	0	0	0	0.04
**Study 8**	0	0.43	0	0	0	0	0.07	0	0.46	0	0	0	0	0.08
**Study 9**	0	0.33	0	0	0	0	0.05	0	0.37	0	0	0	0	0.06
**Study 10**	0.05	0	0.55	0.02	0.01	0	0	0.05	0	0.57	0.02	0.01	0	0
**Study 11**	10.58	0	0.99	3.48	46.54	0.07	0	10.52	0	0.97	3.45	46.50	0.07	0
**Study 12**	0.13	0.07	0.01	0.04	0.03	19.36	0.01	0.13	0.07	0.01	0.04	0.03	19.27	0.01
**Study 13**	0	0.03	0	0	0	0	0.18	0	0.03	0	0	0	0	0.18
**Study 14**	0	0.05	0	0	0	0	0.29	0	0.05	0	0	0	0	0.33
**Study 15**	0	0.03	0	0	0	0	0.18	0	0.03	0	0	0	0	0.18
**Study 16**	0	0.14	0	0	0	0	0.86	0	0.14	0	0	0	0	0.86
**Study 17**	0	0	0	67.13	0	0	0	0	0	0	67.21	0	0	0
**Study 18**	5.82	0	0.54	1.91	33.80	0.04	0	5.78	0	0.53	1.90	33.86	0.04	0
**Study 19**	0.12	0	0.01	0.04	0.68	0	0	0.13	0	0.01	0.04	0.74	0	0
**Study 20**	0.11	0.03	0.01	0.04	0.03	6.72	0	0.13	0.03	0.01	0.04	0.03	8.17	0
**Study 21**	0.51	0.12	0.05	0.17	0.12	31.02	0.02	0.50	0.11	0.05	0.16	0.11	30.44	0.02
**Study 22**	0.77	0.09	0.07	0.25	0.17	0	0.56	1.05	0.12	0.10	0.35	0.24	0	0.76
**Study 23**	0.49	0.06	0.05	0.16	0.11	0	0.35	0.76	0.09	0.07	0.25	0.17	0	0.55
**Study 24**	0.08	0.09	0.01	0.03	0.02	12.07	0.01	0.08	0.08	0.01	0.02	0.02	11.04	0.01
**Study 25**	0.11	0.12	0.01	0.04	0.03	17.24	0.02	0.12	0.13	0.01	0.04	0.03	17.78	0.02
**Study 26**	0.08	0.09	0.01	0.03	0.02	12.55	0.01	0.08	0.09	0.01	0.03	0.02	12.23	0.01
**Study 27**	0	0.14	0	0	0	0	0.36	0	0.14	0	0	0	0	0.37
**Study 28**	0	0.07	0	0	0	0	0.19	0	0.07	0	0	0	0	0.19
**TOTAL**	**100**	**100**	**100**	**100**	**100**	**100**	**100**	**100**	**100**	**100**	**100**	**100**	**100**	**100**
**SUMMARY LOG ODDS RATIO (s.e.)**	-0.161 (0.046)	0.002 (0.032)	-0.044 (0.049)	-0.156 (0.080)	-0.113 (0.062)	-0.197 (0.222)	0.014 (0.039)	-0.165 (0.044)	0.002 (0.030)	-0.045 (0.047)	-0.159 (0.078)	-0.116 (0.060)	-0.196 (0.219)	0.016 (0.037)

* The between-study variance was estimated at 0.000231 for the two-stage analysis (REML), and 0 in the one-stage analysis (ML).
